# Influence of oxygen enriched gases during decompression on bubble formation and endothelial function in self-contained underwater breathing apparatus diving: a randomized controlled study

**DOI:** 10.3325/cmj.2019.60.265

**Published:** 2019-06

**Authors:** Ivana Šegrt Ribičić, Maja Valić, Joško Božić, Ante Obad, Duška Glavaš, Igor Glavičić, Zoran Valić

**Affiliations:** 1 Department of Pulmonary Diseases, University Hospital Center Split, Split, Croatia; 2 Department of Neuroscience, University of Split School of Medicine, Split, Croatia; 3 Department of Pathophysiology, University of Split School of Medicine, Split, Croatia; 4 Department of Health Studies, University of Split, Split, Croatia; 5 Department of Internal Medicine, University of Split School of Medicine, Split, Croatia; 6 Department of Marine Studies, University of Split, Split, Croatia; 7 Department of Physiology, University of Split School of Medicine, Split, Croatia

## Abstract

**Aim:**

To assess the effect of air, gas mixture composed of 50% nitrogen and 50% oxygen (nitrox 50), or gas mixture composed of 1% nitrogen and 99% oxygen (nitrox 99) on bubble formation and vascular/endothelial function during decompression after self-contained underwater breathing apparatus diving.

**Methods:**

This randomized controlled study, conducted in 2014, involved ten divers. Each diver performed three dives in a randomized protocol using three gases: air, nitrox 50, or nitrox 99 during ascent. The dives were performed on three different days limited to 45 m sea water (msw) depth with 20 min bottom time. Nitrogen bubbles formation was assessed by ultrasound detection after dive. Arterial/endothelial function was evaluated by brachial artery flow mediated dilatation (FMD) before and after dive.

**Results:**

Nitrox 99 significantly reduced bubble formation after cough compared with air and nitrox 50 (grade 1 vs 3 and vs 3, respectively, *P* = 0.026). Nitrox 50 significantly decreased post-dive FMD compared with pre-dive FMD (3.62 ± 5.57% vs 12.11 ± 6.82% *P* = 0.010), while nitrox 99 did not cause any significant change.

**Conclusion:**

Nitrox 99 reduced bubble formation, did not change post-dive FMD, and decreased total dive duration, indicating that it might better preserve endothelial function compared with air and nitrox 50 dive protocols.

Self-contained underwater breathing apparatus (SCUBA) diving with compressed air, the most common type of gas used in diving, is associated with venous gas bubbles formation, arterial endothelial dysfunction (1), and other cardiovascular changes (2-4). The excess gas taken up into the tissues during the compression phase of the dive (descent) has to be eliminated when returning to the surface during pressure reduction (decompression). The usage of gas mixture of nitrogen and oxygen (nitrox) with oxygen fractions higher than 21% results in longer no-decompression times for shallow diving and shorter decompression duration for deeper dives (5). Recent data indicate that nitrox use might decrease bubble formation 95 minutes after dive (5). In addition, protocols using pure oxygen during decompression (6,7) and those using oxygen pre-breathing before dives also significantly reduce bubble formation (8,9).

Brachial artery flow-mediated dilatation (FMD) is a standard non-invasive measure for assessing endothelial function (10). It can be transiently decreased as a consequence of repeated breath-hold diving, a single air dive in hyperbaric chamber (1), or at 30 m open sea-water dive (11). Additionally, it may be influenced by exercise, obesity, hormonal status, caffeine, tobacco or alcohol use, and medication (12). Previous studies reported conflicting findings related to the role of oxygen enriched gas mixtures in FMD impairment. Although oxygen enriched gas mixtures decrease bubble formation following in-water diving, they cause a larger decrease in FMD compared with air diving in no-decompression dive to 18 m (13). Partial oxygen pressure of 61 kPa reduced FMD (1), while that of 283 kPa did not affect endothelial function in a simulated dive to 18 m sea water (msw), both in a hyperbaric chamber (14). Furthermore, vascular bubbles were shown to impair endothelial function, indicating that endothelium damage increases with the increasing number of bubbles (1,15).

Although positive effects of oxygen breathing on decreased bubble formation after no-decompression dive have been widely reported, only limited data are available on its effects on both bubble formation and endothelial function following in-water decompression dive. We hypothesized that the use of gas mixtures with increased oxygen level (nitrox 50 and nitrox 99) would reduce bubble formation and preserve FMD. Therefore, the aim of this study was to investigate the changes in bubble formation and FMD in dive protocols using compressed air, nitrox 50, or nitrox 99 during decompression in open-sea field dives at 45 msw in sea-water SCUBA divers.

## Methods

### Study design and participants

This randomized controlled study was conducted in July 2014 at the Big Blue Diving Center in Bol, island of Brač, Croatia. This study involved ten SCUBA divers, five men and five women. Inclusion criteria were experience and knowledge needed to be a licensed SCUBA diver, more than 20 hours of diving per year, and no history of decompression sickness (DCS). Inexperienced and first-time divers, divers younger than 18 years of age, divers with medical conditions such as decompression illness, heart failure, coronary heart disease, malignant disease, and various neurological conditions were excluded from the study. Participants’ average age was 39.3 ± 9.7 years, weight 69.6 ± 13.4 kg, height 173.3 ± 7.1 cm, and body mass index 23.0 ± 0.8 kg/m^2.^ (Table 1). Four divers were smokers, three of them women. The divers restrained from alcohol and tobacco usage for a minimum of 6 hours before the dive.

**Table 1:** Participants’ anthropometric characteristics and diving experience

Parameter (mean ± standard deviation)		Men	Women
Number	10	5	5
Age (years)	39.3 ± 9.7	44.6 ± 12.5	34.0 ± 8.4
Weight (kg)	69.6 ± 13.4	81.2 ± 21.8	58.0 ± 6.7
Height (cm)	173.3 ± 8.0	179.8 ± 49.6	166.8 ± 2.9
Diving experience (years)	17.0 ± 12.8	23.0 ± 12.2	8.2 ± 9.6
Diving experience (hours/year)	151.0 ± 141.5	264.0 ± 136.5	38.2 ± 21.5

All dives were carried out at 9 am or at 3 pm, with the bottom sea water 17°C and outside temperatures ranging from 29-34°C. Each diver performed three dives on three different days, separated with one day break. Each diver served as his/her own control and completed repetitive open-sea dives using compressed air for descent. The diving profiles were limited to 45 msw depth with 20 min bottom time. During ascent to the surface either air, nitrox 50 (50% oxygen and 50% nitrogen), or nitrox 99 (99% oxygen and 1% nitrogen) was used. Each diver performed dives in a randomized (divers drew pieces of paper with dive order from a box) protocol using different gases. In protocol 1 decompression was performed using compressed air; in protocol 2 decompression was performed using nitrox 50; and in protocol 3 decompression was performed using nitrox 99.

**Figure 1:**
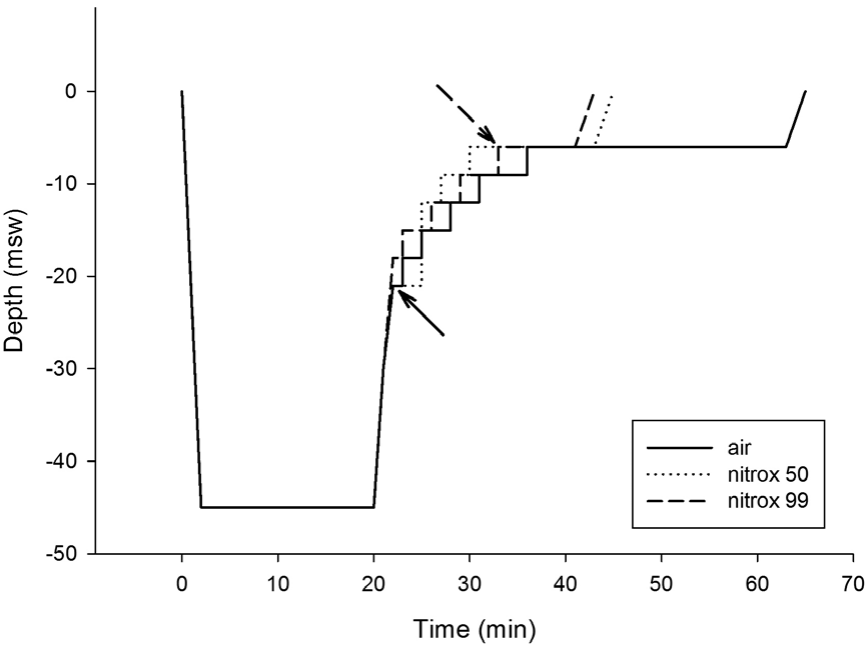
Diving protocols using air, gas mixture composed of 50% nitrogen and 50% oxygen (nitrox 50), or gas mixture composed of 1% nitrogen and 99% oxygen (nitrox 99) during decompression. Indicated are time points when nitrox 50 or nitrox 99 were introduced according to the V planner (nitrox 50 full line arrow, nitrox 99 dashed line arrow).

Nitrox 50 and nitrox 99 were introduced at 21 and 6 msw depth, respectively, according to the V-planner (HHS Software Corp., Kingston, Canada, Figure 1). Thus, total dive duration was 65, 45, and, 43 min for air, nitrox 50, and nitrox 99, respectively. FMD was measured before each dive and 40-60 min after the dive. Venous bubble formation was detected 30 min after each dive at rest and after cough.

All investigational procedures were in accordance with the Declaration of Helsinki. The study was approved by the Research Ethics Committee of the University of Split School of Medicine on May 19th, 2014 (reference number: 2181-198-03-04-14-0023). Possible risks were explained to all participants, who signed the informed consent for study participation and data publication.

### Post-dive monitoring and venous bubble detection

Echocardiographic examination was performed by two experienced cardiologists (DG, AO) thirty minutes post-dive. A phase array probe (1.5-3.3 MHz) of the Vivid Q ultrasonic scanner (GE Healthcare, Milwaukee, WI, USA) was used with the divers supine. Gas bubbles were detected at rest and after two coughs as high intensity echoes in the cardiac chambers. Bubble formation was graded according to the modified scale by Eftedal and Brubakk as follows: 0 = no bubbles, 1 = occasional bubbles, 2 = at least 1 bubble/fourth heart cycle, 3 = at least 1 bubbles/heart cycle, 4 = continuous bubbling, with at least 1 bubble/cm^2^ in all frames, and 5 = “white out,” where individual bubbles cannot be observed (16).

### Endothelial function measurement pre- and post-dive

Endothelial function was assessed by measuring endothelial-dependent flow-mediated vasodilation of the brachial artery following reactive hyperemia with a standard ultrasound procedure (17). The divers were examined in a silent room with a temperature of about 20°C. They rested in supine position on the bench for at least 10 min before measurement was undertaken. Measurements were conducted before the dive and between 40-60 min after surfacing, with a 5.7-13.3 MHz linear transducer using a Vivid Q ultrasonic scanner (GE). Each diver was used as his or her own control for the measurement.

Brachial artery diameter was determined from longitudinal images with the lumen-intima interface positioned on anterior and posterior walls. Images were obtained by means of electrocardiogram (ECG) gating, exploiting the onset of R wave to identify end diastole. After images were selected for analysis, the margins for diameter measurement were detected manually with an electronic caliper. Blood flow velocities were measured by means of a Pulsed Doppler with the sample volume placed in the middle portion of the artery. In order to assure that measurement is performed on the same part of the artery before and after the dive, the location of ultrasound probe was labeled. For most measurements, this corresponded to 3-5 cm proximal to the antecubital fossa. After having acquired the basal measurements, 5 min arterial occlusion was performed by inflating a cuff placed on the forearm to 240 mm Hg. Cuff deflation produced a brief high-flow state, which led to artery dilatation due to increased shear stress. Artery diameter and blood velocity were measured immediately following cuff deflation, at every 30 s for 3 min, and at the fourth and fifth minute. FMD was calculated as the percentage increase in brachial artery diameter from the resting state to maximum dilatation. Raw data were stored on the ultrasound hard drive for later analysis. Since nitroglycerine application can reduce venous bubble formation following decompression, it was not used to determine endothelial-independent dilatation (18).

### Statistical analysis

The number of participants was limited by funding and duration and type of the study. Normality of distribution was tested by Shapiro-Wilk test. Data are presented as mean ± standard deviation or median and range (25%-75% quartile range, and 10%-90% range), where appropriate. Bubble grades data were compared by using the nonparametric Friedman repeated measures analysis of variance, with Tukey test for all pairwise multiple comparisons. FMD data are presented as mean percent change ± standard deviation. Parameters of single type of dive (air, nitrox 50, nitrox 99) were compared by using the *t* test for paired samples (predive vs postdive). Parameters for the three types of dive were analyzed by two way repeated measures ANOVA (type of dive [air, nitrox 50, nitrox 99] × time [pre-dive, post-dive]), with Tukey test for pairwise multiple comparisons. The significance level was set at *P* < 0.05. Statistical analysis was performed using SigmaPlot 12.5 software (Systat Software, San Jose, CA, USA).

## RESULTS

### Dive duration

All divers successfully completed the specified protocol without DCS symptoms. In air, nitrox 50, and nitrox 99 dives, diving to 45 msw with 20 minutes bottom time lasted 65, 45, and 43 minutes, respectively (Figure 1). The exposure to hyperoxic gas mixture was 0, 20, and 8 min, respectively.

### Bubble grade

**Figure 2:**
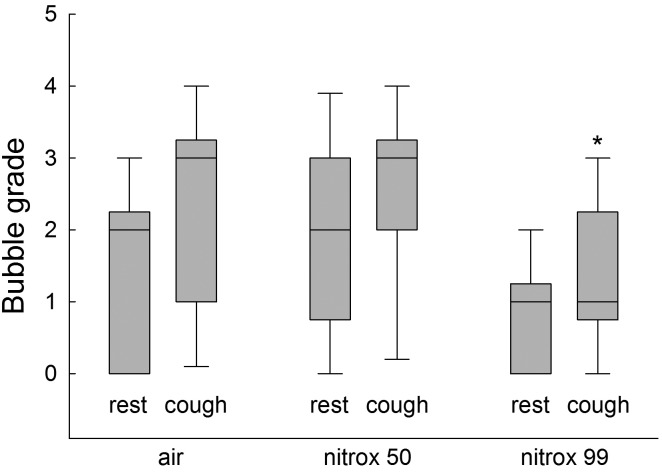
Venous gas bubble formation after air, gas mixture composed of 50% nitrogen and 50% oxygen (nitrox 50), or gas mixture composed of 1% nitrogen and 99% oxygen (nitrox 99) dive before (rest) and after cough (cough). Bubble formation was higher 30 minutes after dive for protocols using air and nitrox 50 compared with nitrox 99 after cough. Data are presented as medians (horizontal lines), 25th and 75th quartiles (shaded squared areas), and 10th to 90th ranges (error bars) of the venous bubble grades measured after the dives. Asterisk indicates values significantly different from nitrox 50 (Friedman analysis of variance, *P* < 0.05).

Bubble grade assessment 30 minutes after dive for air, nitrox 50, or nitrox 99 showed the grade of 2, 2, and 1, respectively, at rest (before cough) (Friedman repeated measures analysis of variance, *P* = 0.114; Figure 2). Air and nitrox 50 protocol significantly increased bubble formation after cough compared with nitrox 99 (grade of 3, 3, and 1, respectively, Friedman repeated measures analysis of variance, *P* = 0.026; Figure 2).

### Flow mediated dilatation

**Figure 3:**
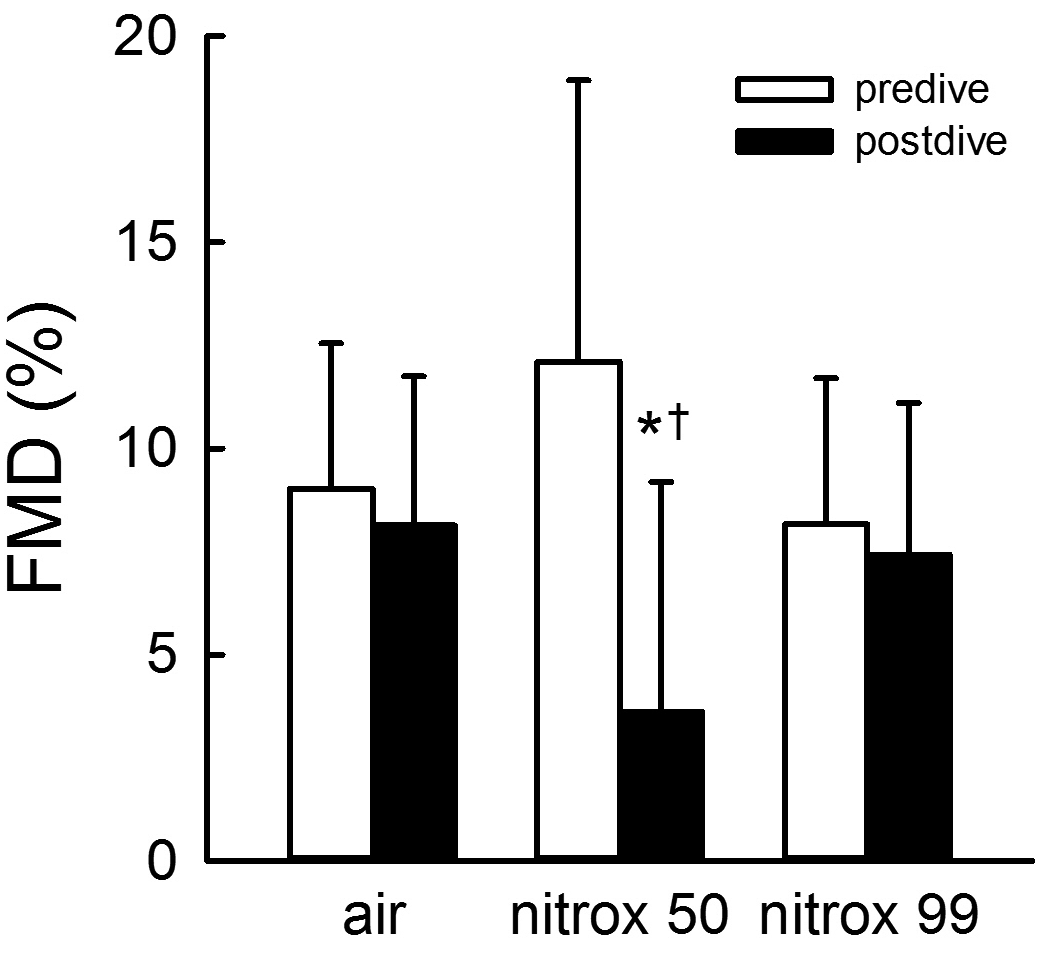
Flow mediated vasodilatation (FMD) was significantly decreased in gas mixture composed of 50% nitrogen and 50% oxygen (nitrox 50) diving protocol. FMD values are shown before and after dives for air, nitrox 50, and gas mixture composed of 1% nitrogen and 99% oxygen (nitrox 99) dives. Asterisk indicates values significantly different from nitrox 50 pre-dive. *t* test for paired samples; dagger indicates values significantly different from air post-dive, two-way repeated measures ANOVA, *P* < 0.05). Open – pre-dive; closed – post-dive.

**Figure 4:**
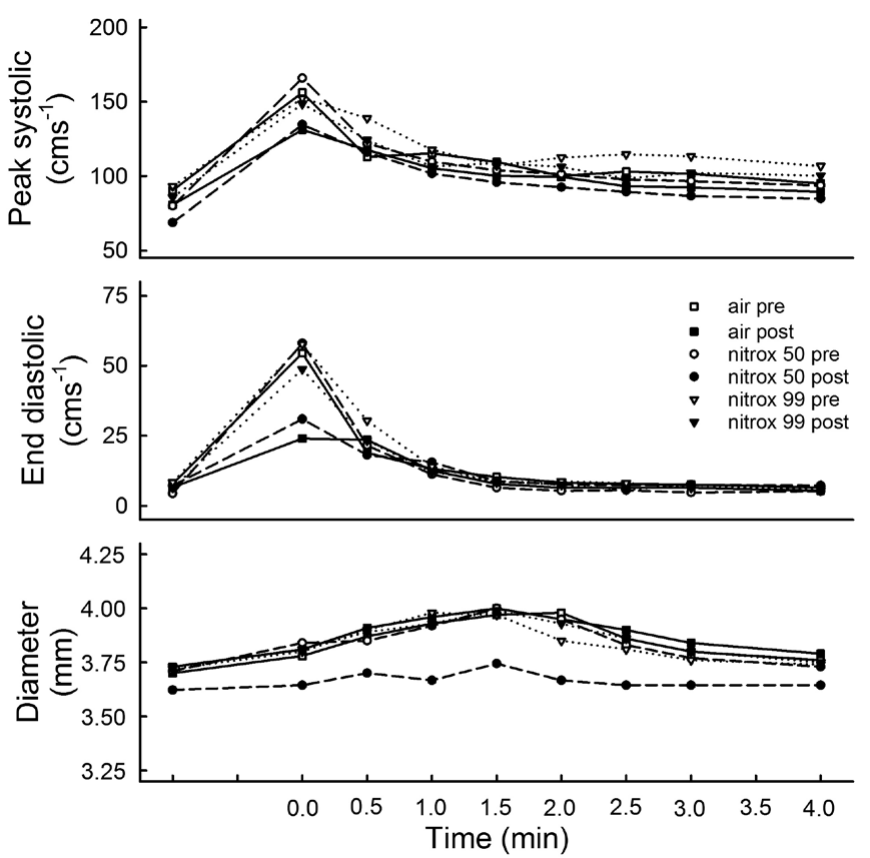
Absolute values of peak systolic blood velocities, end-diastolic blood velocities, and brachial artery diameter before and during 4 minutes after the release of the underarm cuff. Open square – air before; closed square – air after; open circle – nitrox 50 before; closed circle – nitrox 50 after; open triangle – nitrox 99 before; closed triangle – nitrox 99 after.

In air and nitrox 99 dives, there was no significant difference between pre- and post-dive FMD (in air: 9.03 ± 3.52% vs 8.14 ± 3.61%, *t* test for paired samples, *P* = 0.204; and in nitrox 99: 8.17 ± 3.54% vs 7.43 ± 3.68%, respectively, *t* test for paired samples, *P* = 0.373; Figures 3 and 4), but in nitrox 50 dive FMD was significantly decreased post-dive (12.11 ± 6.82% vs 3.62 ± 5.57%, *t* test for paired samples, *P* = 0.010; Figure 3). Nitrox 50 significantly decreased post-dive FMD compared with air (3.62 ± 5.57% vs 8.14 ± 3.61% respectively, two way repeated measures ANOVA with Tukey test, *P* = 0.031; Figure 3). There were no differences in peak systolic, end-diastolic blood velocities, and brachial artery diameter (Figure 4).

## DISCUSSION

Nitrox 99 protocol significantly decreased bubble formation after cough compared with air and nitrox 50 protocols. When it comes to the effects on FMD, although in air and nitrox 99 dives, there was no significant difference between post-dive FMD and pre-dive FMD, nitrox 50 significantly decreased post-dive FMD. Furthermore, nitrox 50 dives had 20 minutes shorter decompression duration than air dives, but the two protocols did not differ in bubble formation. Changes in peak systolic velocities, end diastolic velocities, and brachial artery diameters were not observed.

Recent studies in no-decompression diving profiles at 18 msw showed less venous bubbling in nitrox dives compared with air dives (13). Our results are in accordance with the previous research showing reduced bubble formation in a nitrox 99 protocol (6). Such reduction was significant only after cough, although it also appears to be present during rest. The observed difference might be explained by increased mobilization of gas bubbles during coughing or limb movement (5-7,13).

During SCUBA diving, bubbles might be created from pre-existing gas micronuclei due to the excess nitrogen dissolved under increased hydrostatic pressure (19,20). Nitrogen from tissues can be washed out by denitrogenation procedure. In this procedure, higher partial pressure of inspired oxygen increases arterial oxygen pressure and increases dissolved nitrogen diffusion from tissues into the blood. Tissue nitrogen saturation is reduced, thus limiting bubble formation. Indeed, it seems that breathing oxygen before diving might eliminate gas micronuclei before they create bubbles (8,21). Additionally, pretreatment with hyperbaric oxygenation (HBO) better reduces decompression-induced bubble formation compared with normobaric oxygenation, probably because it provides more oxygen to the whole body to replace nitrogen (22).

In our study, since nitrox 50 dive decreased post-dive FMD, we expected that nitrox 99 will decrease it even more. However, nitrox 99 dive preserved post-dive FMD. Previous studies have shown considerable variability in FMD response, indicating an FMD reduction (1,13) following dives with gas mixtures with increased oxygen level. The conflicting results can be explained by different methodological approaches, which can limit the comparability of the outcomes.

Hyperoxia is an important issue in diving since inspired partial oxygen pressure is increased compared with breathing air at normobaric pressure. It was reported to have both beneficial and deleterious effects, with the latter increasing progressively with the increased inspired partial pressure and longer exposure duration. Hyperoxia decreased FMD in individuals who received standard HBO protocol (240 kPa oxygen) (23) and decreased cell viability and proliferation in isolated human microvascular endothelial cells (24).

Hyperoxia may lead to vasoconstriction, but the exact mechanisms underlying this process remain unclear. It may also decrease NO levels, which is crucial for endothelium-driven vasodilatation and vascular homeostasis, attenuating FMD (1,24). Several studies have concluded that brachial and radial FMD depend on a NO pathway, the blockade of which abolished the dilatory response in young healthy humans (25). Another possible pathway is the hyperoxia-induced decline of cyclooxygenase activity in endothelial cells, decreasing vasodilatory prostaglandins levels (24,26). The increased production of reactive oxygen species is also important for the effect of hyperoxia on cell viability and vascular tone (24,27).

On the other hand, hyperbaric oxygen therapy as a support to conventional therapy increases FMD and NO production, as well as calcitonin gene-related peptide in patients with slow coronary flow (28). Our study was not designed to clarify the mechanism underlying the observed changes, but it is possible that hyperoxic exposure duration have played an important role in the FMD outcomes. Thus, the rise in arterial oxygen pressure during 20 minutes of nitrox 50 breathing might have attenuated FMD, while 8-minute hyperoxic exposure when breathing nitrox 99 might have preserved FMD.

Although changes in arterial endothelial function can occur without the direct contact with the bubbles, larger FMD reduction occurs in divers with higher bubble grade (1). In our study, significantly lower bubbling in nitrox 99 dive might be related to FMD preservation compared with nitrox 50 dive, although there was no significant association between the observed bubble grade (at rest or cough procedure) and FMD measured after the dive.

Repetitive exposures to increased oxygen levels may have opposing effects and are often associated with acute lung injury, enlarged infarct size, and increased mortality in patients with cardiac arrest (29-31). Additionally, hyperoxia-induced ROS production was linked to cancer, diabetes mellitus, cardiovascular diseases, and aging (27). In contrast, other studies report improved organ function, anti-inflammatory effects, and antibacterial action (24,27). Additionally, increased partial oxygen pressure might cause seizures in SCUBA divers and patients undergoing HBO therapy due to CNS oxygen toxicity (27,32,33). However, despite different gas mixtures and relatively deep dive in this study, divers did not experience any symptoms related to CNS toxicity.

The study results have to be interpreted in light of some limitations. First, the study was not designed to explain the mechanism of the FMD reduction. Additionally, it involved a limited number of participants who were their own controls. The age range of 25-56 years might have affected the results since hyperoxia influences endothelial function differently in younger and older people, possibly because older individuals have pre-existing reduced vascular compliance (23). Furthermore, we did not determine the menstrual cycle phase in female participants, although FMD varies during menstrual cycle and is associated with changes in serum estradiol concentration (34).

We are aware that the use of air for diving to depths greater than 40 msw carries a certain risk of DCS development, and high nitrogen and oxygen contents could cause nitrogen narcosis and cerebral oxygen toxicity. In our study, no divers experienced DCS either during the study or in the past.

Nitrox 99 dive protocol had the shortest dive duration and did not significantly change post-dive FMD, suggesting that it can preserve endothelial function. These results might elucidate the impact of air enriched gas mixtures on FMD and bubble formation, which should be taken into consideration when deciding about diving protocols.
